# Rare bilateral vascular variations of the upper limb: a cadaveric case study

**DOI:** 10.1186/s13019-024-03158-z

**Published:** 2024-12-27

**Authors:** Ricky Smith, Yasith Mathangasinghe, David Gonsalvez

**Affiliations:** Centre for Human Anatomy Education, Department of Anatomy and Developmental Biology, Biomedical Discovery Institute, Faculty of Medicine, Nursing and Health Sciences, Monash University, Clayton, Australia

**Keywords:** Arterial variations, Vascular anomaly, Superficial ulnar artery, Axillary artery, Cadaveric

## Abstract

Arterial variations in the upper limb are of significant clinical importance, especially in procedures such as venepunctures, coronary artery bypass grafts, trauma reconstructive surgeries, brachial plexus nerve blocks, and breast reconstructions. This report presents previously undocumented arterial variations in the upper limbs in a 95-year-old female cadaveric donor. We observed bilateral superficial ulnar arteries originating at the cubital fossa, deviating from the previously reported origin at the proximal brachial artery. We found additional variations in the branches of the axillary artery: on the right side, two superior thoracic arteries emerged from the first part of the axillary artery, an accessory branch supplied the subscapular muscle, and the large subscapular artery arising from the third part of the axillary artery gave rise to both the lateral thoracic and posterior circumflex humeral arteries. On the left side, a common trunk was identified, giving rise to the transverse cervical, dorsal scapular, and accessory lateral thoracic and subscapular arteries. Moreover, the acromial artery originated directly from the axillary artery on both sides. This case report discusses the clinical significance of these unique vascular anatomical variants, their prevalence, and potential impact, emphasizing the importance for clinicians to be aware of such variations to enhance surgical planning and patient safety.

## Introduction

Arterial variations in the upper limb are common and have significant implications for clinical practice, especially in surgical and diagnostic procedures. While deviations from the typical branching patterns of the axillary artery are observed in over 80% of cases, rare variations such as the superficial ulnar artery can pose unique challenges. Here, we report a case of multiple rare arterial variations observed in a 95-year-old assigned female at birth cadaveric donor, including bilateral superficial ulnar arteries originating from the cubital fossa and atypical branching patterns of the axillary artery. These findings highlight the importance of understanding and identifying such variations to enhance surgical accuracy and patient safety.

## Case presentation

**Fig. 1:**
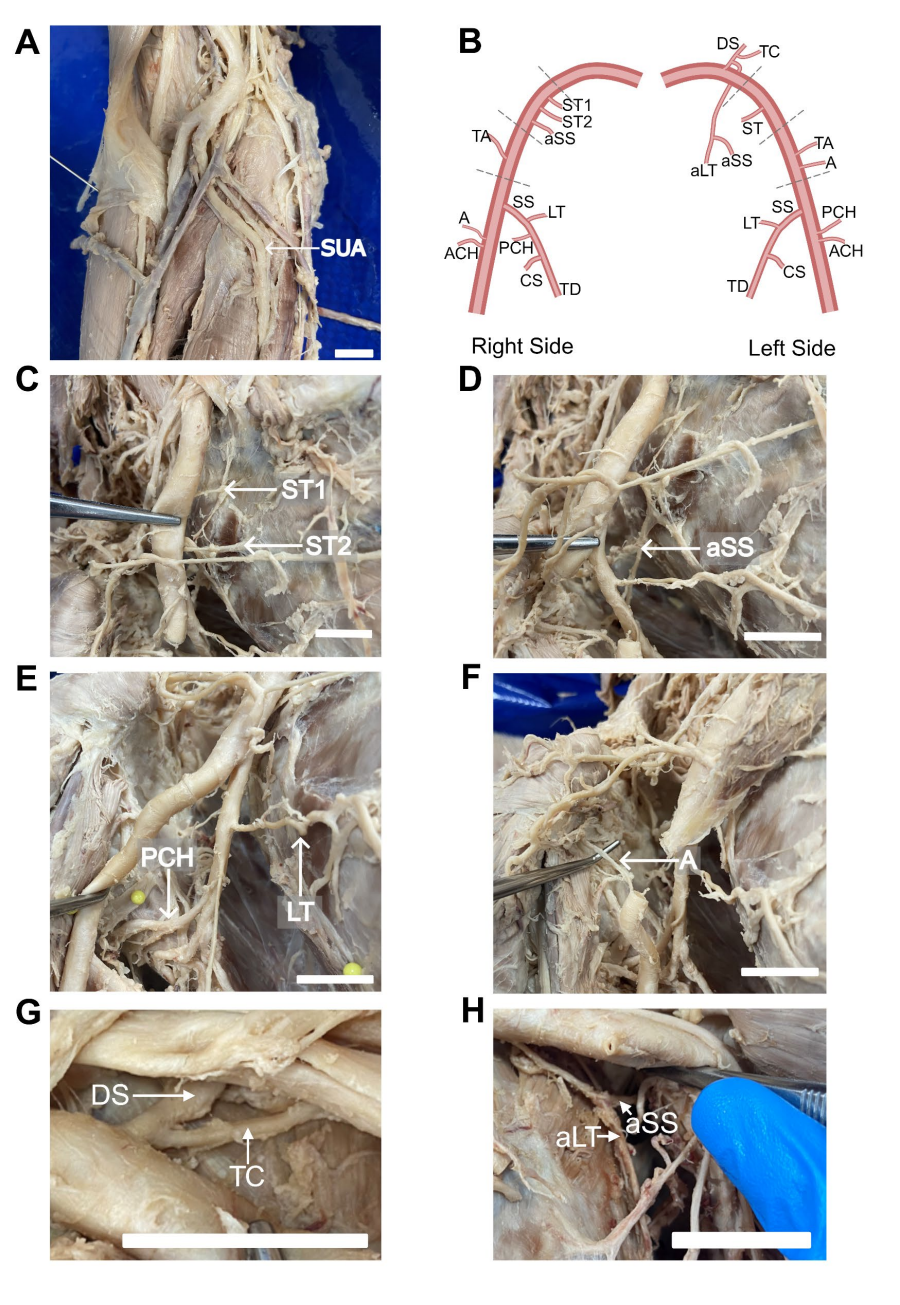
Anatomical variations in upper limb arterial supply. **A**, The superficial ulnar artery (SUA) arising from the brachial artery in the cubital fossa. **B**, Illustration of the anatomical variations of the arterial branches of the axillary artery. ST1, first branch of superior thoracic artery; ST2, second branch of superior thoracic artery; aSS, accessory subscapular artery branch; TA, thoracoacromial artery; SS, subscapular artery; LT, lateral thoracic artery; PCH, posterior humeral circumflex artery; CS, circumflex scapular artery; TD, thoracodorsal artery; A, acromial branch; ACH, anterior humeral circumflex artery; DS, dorsal scapular artery; TC, transverse cervical artery; aLT, accessory lateral thoracic artery. **C.** Two superior thoracic artery branches (ST1, ST2) arising from the axillary artery. **D**, accessory subscapular artery (aSS). **E**, posterior circumflex humeral artery (PCH) and lateral thoracic (LT) arising from the subscapular artery. **F**, acromial branch (A) arising from the axillary artery. G, dorsal scapular (DS) and transverse cervical (TC) branches arising from a common trunk. H, accessory subscapular (aSS) branch that originated from the common trunk giving rise to an accessory lateral thoracic artery (aLT). Scale bar = 2 cm

We conducted a routine dissection of a 95-year-old, 55 kg assigned female at birth, who died of ischemic heart disease. A superficial ulnar artery was observed in the donor’s right upper limb (Fig. 1A). This superficial ulnar artery was smaller in calibre than the radial artery and initially dived deep into the proximal third of the forearm between the flexor carpi radialis and palmaris longus before following the typical course. There were no branches of the ulnar artery in the forearm. Tracing the arterial vessels, we discovered that the radial artery gave rise to the common interosseous artery. The bilateral occurrence of superficial ulnar artery was confirmed by dissecting the contralateral limb.

We traced the arterial supply of both upper limbs proximally to investigate further arterial variations. Multiple variations of the axillary artery were found (Fig. 1B). In the right upper limb, two separate branches of the superior thoracic artery arose from the first part of the axillary artery (Fig. 1B, C). An accessory branch from the first part of the axillary artery supplied the subscapular muscle (Fig. 1B, D). In the third part of the axillary artery, a large-calibre subscapular artery gave rise to both the lateral thoracic artery and the posterior circumflex humeral artery (Fig. 1B, E). Additionally, the acromial branch arose directly from the AA rather than from the thoracoacromial trunk (Fig. 1B, F).

In the left upper limb, a common trunk gave rise to the transverse cervical artery, the dorsal scapular artery, and a proximal branch that gave rise to both an accessory lateral thoracic artery and an accessory subscapular artery (Fig. 1B, G, H). Similar to the right upper limb, the acromial artery originated directly from the axillary artery rather than the thoracoacromial artery. Furthermore, the lateral thoracic artery arose as a branch of the subscapular artery rather than directly from the second segment of the axillary artery, which is its classic origin.

## Discussion

This is the first documented case of multiple bilateral upper limb arterial vascular variations associated with bilateral superficial ulnar arteries. Notably, the arterial variations differed between the right and left upper limbs, illustrating the considerable variability in upper limb vascular anatomy.

### Superficial ulnar artery

The prevalence of superficial ulnar artery varies in the literature, with an estimated incidence between 0.7 and 9.4% [1]. Importantly, this is the first documented case of bilateral superficial ulnar arteries originating from the cubital fossa, which contrasts with the more proximal origin seen in superficial brachioulnar artery variations [2]. Rodríguez-Niedenführ et al. introduced the term superficial brachioulnar artery to describe an ulnar artery with a high origin that courses superficially over the forearm flexor muscles. In their study of 192 cadavers, superficial brachioulnar arteries were found in 10 (5.2%) cases, and all assigned female at birth cadavers with superficial brachioulnar arteries had this variation bilaterally, consistent with our findings. Although a similar case of superficial ulnar artery originating at the cubital fossa has been reported, it was unilateral in an assigned male at birth cadaver [3]. In our case, the common interosseous arteries arose from the radial arteries bilaterally, a variation frequently observed alongside superficial ulnar arteries [4–13].

### Axillary artery branches

Variations in the branching pattern of the axillary artery are common, but the combination observed in this case, along with accessory vessels, is unique. Variations in the branches of the second and third parts of the axillary artery are more common, while variations in the first part are less frequently reported [14].

Of the variations observed in this case, a variant lateral thoracic artery is the most common, with the classic origin occurring in only 40-70.3% of cases [14]. Thiele et al. reported the lateral thoracic artery originating from the thoracodorsal artery in 37% of cases and from the subscapular artery in 21% of cases, the latter of which occurred bilaterally in our case. The posterior circumflex humeral artery originating from the subscapular artery, as observed unilaterally in our case, is a common variant, occurring in 19.4% of cases [14]. The tetrafurcation of the subscapular artery observed in the right upper limb of our case has also been previously reported [15, 16].

To the best of our knowledge, this is the first report of dual superior thoracic arteries. These vessels in the present case occurred unilaterally on the right side, were of small calibre, and originated adjacent to each other at the classic origin in the first part of the axillary artery. Variation in the first part of the axillary artery is a very rare finding [17]. Additionally, the origin of the superior thoracic artery in this segment is typically constant, with a prevalence of 97% [14].

Variation of the thoracoacromial artery and its classic terminal branches, the pectoral, deltoid, clavicular and acromial have been described in the literature. Commonly, the pectoral and deltoid branches are constant, whilst the clavicular and acromial branches are variable [18]. Bonczar et al. describe the acromial artery as the least common of the four typical thoracoacromial artery to arise from its this direct origin, and in fact more commonly originates from the deltoid branch of the thoracoacromial artery. The variant acromial branch origin directly from the axillary artery bilaterally described in our case adds further evidence to the high variability of this arterial branch.

Accessory subscapular arteries are a very rare variant. Origin of this anomalous vessel in the first segment of the axillary artery at the level of the superior thoracic artery as observed in the right upper limb of our case has been previously described in the literature, noted as the “muscular branch 1” [19].

### Subclavian artery branches

Variations in the branching patterns of the subclavian artery are relatively common, yet the finding of a common trunk giving rise to the dorsal scapular artery, transverse cervical artery, accessory lateral thoracic artery and accessory subscapular artery is particularly unique. While accessory subscapular artery and accessory lateral thoracic arteries are rare, their origin from a common trunk at the distal segment of the subclavian artery has not been previously documented in the literature. An accessory or duplicated lateral thoracic artery is known to occur with a prevalence of approximately 6% [20], with reported origins from either the axillary artery or the superior thoracic artery [20]. However, no prior studies describe an accessory lateral thoracic branch originating from the distinctive subclavian trunk observed in this case.

### Clinical implications

Understanding the extensive anatomical variations of upper limb vasculature is crucial for surgeons and radiologists to ensure the safe execution of procedures and accurate interpretation of angiography. This case highlights the potential for multiple coexisting arterial variations within a single individual.

Superficial ulnar artery pose a risk of iatrogenic injury during procedures such as cubital fossa cannulation or venepuncture, as documented in the literature [6, 11, 21]. There have been reports of accidental intra-arterial cannulation due to this anomaly [22]. Additionally, the smaller calibre of the ulnar artery compared to the radial artery may impact collateral blood supply to the hand, potentially complicating procedures such as radial artery coronary bypass grafts or radial forearm flaps, especially in cases with bilateral superficial ulnar arteries as described in this report. Nevertheless, superficial ulnar artery fasciocutaneous flaps have been used successfully as an alternative to radial forearm flaps, particularly when Allen’s tests is positive [23].

Typically, superficial ulnar arteries originate more proximally from either the brachial or axillary artery. Awareness of the possibility of a superficial ulnar artery at the classical bifurcation level in the cubital fossa is essential to minimize the risk of iatrogenic injury and enhance procedural planning. On this note, the presence of a common interosseous artery arising from the radial artery as a paired variation with superficial ulnar artery can aid in more accurate angiographic interpretation and further reduce iatrogenic risk.

Variations in the branching patterns of the axillary and subclavian arteries are significant for clinicians due to the range of interventions performed in this region. Knowledge of these variations is relevant for procedures involving the axilla, such as trauma reconstruction, brachial plexus anaesthesia blocks, coronary artery bypass grafts, and breast reconstruction [14]. Particularly, the presence of acromial arteries originating directly from the axillary artery is particularly pertinent for thoracoacromial perforator flap procedures [18, 24, 25]. Specifically, the tetrafurcation of the subscapular artery is clinically challenging for procedures such as flap harvesting in this region [15, 16].

In summary, we present a unique constellation of novel and rare vascular variants in the upper limb. Awareness of these variants is crucial for clinicians to minimize the risk of iatrogenic vascular injuries and to potentially improve outcomes in regional surgical interventions.

## Data Availability

No datasets were generated or analysed during the current study.

## References

[1] Dartnell J, Sekaran P, Ellis H. The superficial ulnar artery: incidence and calibre in 95 cadaveric specimens. Clin Anat. 2007;20(8):929–32. 17907204 10.1002/ca.20546

[2] Rodríguez-Niedenführ M, et al. Variations of the arterial pattern in the upper limb revisited: a morphological and statistical study, with a review of the literature. J Anat. 2001;199(Pt 5):547–66. 11760886 10.1046/j.1469-7580.2001.19950547.x PMC1468366

[3] Kumka M, Purkiss S. A rare case of unilateral variations of forearm arteries: anatomy, embryology and clinical implications. J Can Chiropr Assoc. 2015;59(3):253–60. 26500359 PMC4593037

[4] Sieger J, et al. Superficial brachioulnar artery and its clinical significance. Anat Cell Biol. 2019;52(3):333–6. 31598363 10.5115/acb.19.008 PMC6773909

[5] Baral P, et al. Multiple arterial anomalies in upper limb. Kathmandu Univ Med J (KUMJ). 2009;7(27):293–7. 20071879 10.3126/kumj.v7i3.2740

[6] Bhat KM, Potu BK, Gowda S. High origin of ulnar artery in south Indian male cadaver: a case report. Rom J Morphol Embryol. 2008;49(4):573–5. 19050810

[7] Chunder R, Mukherjee K, Guha R. An unusual ulnar artery: its embryological basis and clinical significance. J Indian Med Assoc. 2011;109(12):934–5. 23469581

[8] Clarke E, et al. Anatomical variations of the superficial ulnar artery: case series observed on historical specimens prepared by Ludwik Karol Teichmann. Folia Morphol (Warsz). 2022;81(1):227–33. 33577075 10.5603/FM.a2021.0014

[9] Gupta G, et al. Bilateral superficial ulnar artery with high origin from the axillary artery: its anatomy and clinical significance. Folia Morphol (Warsz). 2012;71(1):48–51. 22532186

[10] Shyamala G, et al. Bilateral arterial variation in the upper extremity–an anatomical case report. Clin Ter. 2013;164(6):523–5. 24424217 10.7417/CT.2013.1631

[11] Vollala VR, Jetti R, Soni S. High origin of an ulnar artery–development and surgical significance. Chang Gung Med J. 2011;34(6 Suppl):39–42. 22490457

[12] Yazar F, et al. An unusual variation of the superficial ulnar artery. Surg Radiol Anat. 1999;21(2):155–7. 10399219 10.1007/s00276-999-0155-1

[13] Hansdak R, et al. Unusual branching pattern of brachial artery - embryological basis and clinicoanatomical insight. Clin Ter. 2015;166(2):65–7. 25945432 10.7417/CT.2015.1817

[14] Thiele CM, et al. Axillary artery variation: the rule not the exception. Natl J Clin Anat. 2020;9(3):82–9.

[15] Dimovelis I, et al. Tetrafurcation of the subscapular artery. Anatomical and clinical implications. Folia Morphol (Warsz). 2017;76(2):312–5. 27714732 10.5603/FM.a2016.0057

[16] Hadimani S, et al. Fenestration of axillary vein by a variant axillary artery. Kathmandu Univ Med J (KUMJ). 2013;11(42):162–4. 24096226 10.3126/kumj.v11i2.12494

[17] Brilakis L, et al. Prevalence of Axillary Artery variants and their clinical significance: a scoping review. Cureus. 2023;15(10):e47809. 38021835 10.7759/cureus.47809 PMC10679784

[18] Bonczar M, et al. The thoracoacromial trunk: a detailed analysis. Surg Radiol Anat. 2022;44(10):1329–38. 36094609 10.1007/s00276-022-03016-4 PMC9649491

[19] Oza PP, Yung HC, Vaz K, Grace. Variations in the branching pattern and course of the Left Axillary artery: a cadaveric case report. Cureus. 2023;15(6):e40852. 37489202 10.7759/cureus.40852 PMC10363335

[20] Olinger A. *Upper Limb Arteries*, in *Bergman’s Comprehensive Encyclopedia of Human Anatomic Variation*. 2016. pp. 583–618.

[21] Casal D, et al. A rare variant of the ulnar artery with important clinical implications: a case report. BMC Res Notes. 2012;5:660. 23194303 10.1186/1756-0500-5-660 PMC3529700

[22] Chin KJ, Singh K. The superficial ulnar artery–a potential hazard in patients with difficult venous access. Br J Anaesth. 2005;94(5):692–3. 15814810 10.1093/bja/aei548

[23] Bell RA, Schneider DS, Wax MK. Superficial ulnar artery: a contraindication to radial forearm free tissue transfer. Laryngoscope. 2011;121(5):933–6. 21520105 10.1002/lary.21465

[24] Portenard AC, et al. Anatomical study of the perforator flap based on the acromial branch of the thoraco-acromial artery (abTAA flap): a cadaveric study. Surg Radiol Anat. 2019;41(11):1361–7. 31493006 10.1007/s00276-019-02322-8

[25] Kodaira S, Fukumoto K, Kato N. Free thoracoacromial artery Perforator Flap for skin defects of the dorsal hand. Tech Hand Up Extrem Surg. 2018;22(2):68–71. 29664802 10.1097/BTH.0000000000000192

